# Elucidating the Water and Methanol Dynamics in Sulfonated Polyether Ether Ketone Nanocomposite Membranes Bearing Layered Double Hydroxides

**DOI:** 10.3390/membranes12040419

**Published:** 2022-04-13

**Authors:** Ernestino Lufrano, Isabella Nicotera, Apostolos Enotiadis, Muhammad Habib Ur Rehman, Cataldo Simari

**Affiliations:** Department of Chemistry and Chemical Technologies, University of Calabria, 87036 Rende, Italy; ernestino.lufrano@unical.it (E.L.); isabella.nicotera@unical.it (I.N.); aenotiadis@gmail.com (A.E.); engr.habib952@gmail.com (M.H.U.R.)

**Keywords:** direct methanol fuel cells, sPEEK, layered double hydroxides, nanocomposite membranes, PFG-NMR, methanol crossover, proton conduction, ion selectivity

## Abstract

Conventional Nafion membranes demonstrate a strong affinity for methanol, resulting in a high fuel crossover, poor mechanical stability, and thus poor performance in direct methanol fuel cells (DMFCs). This study involves the synthesis and physiochemical characterization of an alternative polymer electrolyte membrane for DMFCs based on sulfonated poly(ether ether ketone) and a layered double hydroxide (LDH) material. Nanocomposite membranes (sPL), with filler loading ranging between 1 wt% and 5 wt%, were prepared by simple solution intercalation and characterized by XRD, DMA, swelling tests, and EIS. For the first time, water and methanol mobility inside the hydrophilic channels of sPEEK-LDH membranes were characterized by NMR techniques. The introduction of LDH nanoplatelets improved the dimensional stability while having a detrimental effect on methanol mobility, with its self-diffusion coefficient almost two orders of magnitude lower than that of water. It is worth noting that anionic lamellae are directly involved in the proton transport mechanism, thus enabling the formation of highly interconnected paths for proton conduction. In this regard, sPL_3_ yielded a proton conductivity of 110 mS cm^−1^ at 120 °C and 90% RH, almost attaining the performance of the Nafion benchmark. The nanocomposite membrane also showed an excellent oxidative stability (over more than 24 h) during Fenton’s test at 80 °C. These preliminary results demonstrate that an sPL_3_ nanocomposite can be potentially and successfully applied in DMFCs.

## 1. Introduction

PEM (polymer electrolyte membrane) fuel cells have now gained global attention as a next generation green electrochemical energy generator due to their high efficiencies and low operating temperatures [1].

Although renewable energy has been exploited rapidly in recent years to alleviate the depletion of fossil fuel reserves and the pressure of environmental protection, most of them are unstable and intermittent during generation, and thus these valuable electric energies are difficult to apply continuously and stably.

Among all PEM fuel cell devices, direct methanol fuel cells are quickly becoming the primary candidates for practical applications, both in stationary devices and automobiles. Compared to the more well-known hydrogen-fueled PEMFCs, DMFCs obviously exhibit higher CO_2_ emissions but also present several intriguing advantages, including the use of a liquid, low-cost, and easy to handle fuel for power generation, a high energy density (6.1 kWh Kg^−1^), easy fuel recharging, and a simple system design [2,3,4]. Indeed, in DMFCs there is no need for fuel reforming and/or onboard hydrogen storage. This obviously decreases the technology cost [5]. Similarly, DMFCs also possess clear advantages over redox flow batteries, i.e., another type of zero-emission power system, which suffer from strong power and energy density limitations and are typically based on the use of toxic, corrosive, and expensive fluids [6].

The polymer electrolyte is one of the key components in DMFC assembly as it acts as a gas separator between two electrodes as well as a pathway for ion exchange [7]. Since the 1960s, Nafion has been proved and applied as an effective proton exchange electrolyte material for many electrochemical devices due to its high ion conductivity, chemical, mechanical, and thermal stability, and long life [8]. Despite all the advantages of Nafion membranes, its use in direct methanol fuel cells (DMFCs) is affected by some limitations. The main one is surely the fuel permeation from the anode side to the cathode, which reduces the conversion efficiency due to fuel wasting, catalyst poisoning, and mixed potential [9,10]. Moreover, the cost of Nafion membranes is still very high (5000 €/kg) [11]. To overcome the drawbacks of Nafion membranes, two major strategies have been adopted by researchers over the years. First is the incorporation of some organic or inorganic fillers with Nafion^®^ to hinder the methanol crossover. Many studies have been conducted on incorporating different new materials, such as graphene oxide [12,13], silica [14], nanofibers [15], carbon nanotubes (CNTs) [16], and eggshell [17], with Nafion to enhance its physiochemical properties. However, it has mostly been reported that the combination of Nafion with nanocomposites does not influence the fuel crossover a great deal, which is due to the high affinity of Nafion for methanol [18]. Against this background, the development of novel membranes based on non-fluorinated polymers seems to be the most promising approach toward the development of PEMs with a low methanol permeability. Therefore, in recent years, a large number of polyaromatic ionomers have been widely explored to avoid the demerits of Nafion, including sulfonated poly(arylene ether ketone) [19,20], sulfonated polyimide [21,22], and sulfonated polysulfone [23,24]. Among these, inexpensive sulfonated poly(ether ether ketone) is clearly one of the most promising electrolytes for DMFCs since it combines high mechanical, thermal, and chemical stability with an inherently low affinity toward methanol [25,26,27]. The introduction of sulfonic acid groups into the polymer backbone by sulfonation reactions is the most common strategy to achieve a satisfactory hydrophilicity and good proton conduction in the membrane. Following the sulfonation, in fact, sPEEK can be assimilated to a copolymer comprising hydrophobic non-sulfonated PEEK structural units and sulfonated PEEK units which are hydrophilic. This peculiar structure provides a peculiar microphase segregation which simulates that of Nafion: the mechanical strength is ensured by the non-sulfonated units while the sulfonated polar clusters can absorb significant amounts of water, thus enabling proton conduction [28]. Unfortunately, satisfactory conductivity performances are typically achieved at a high sulfonation degree, with a detrimental effect on the mechanical strength and on the methanol resistance [29]. As a combined approach, the preparation of inorganic–organic hybrid membranes, where inorganic nanoparticles are finely dispersed into a polymeric proton-conducting matrix, has been widely explored to increase proton conductivity while simultaneously increasing the methanol resistance of the electrolyte [30]. Consequently, a large number of fillers, such as titania, zirconia, silica, and zeolite particles [31,32,33,34,35], have been tested to reduce methanol crossover and enhance water retention though, in most cases, these composite PEMs, which exhibit a lower proton conductivity than the parental polymer. Indeed, the strong interaction between the inorganic nanoparticles and the hosting polymer alters the nanophase segregation of the resulting PEM by reducing the size of its ionic clusters. This decreases the fraction of bulk water and, in turn, has a detrimental effect on ion mobility [36]. Instead, we have recently demonstrated that the introduction of functionalized 2D-layered materials, such as graphene oxide, siliceous layered materials smectite clay, and layered double hydroxides, is able to successfully enhance the mechanical resistance, hydrophilicity, and proton conductivity of the resulting electrolyte, while simultaneously improving its methanol resistance [37,38,39,40,41,42].

Among these class of materials, layered double hydroxides (LDHs) have been extensively investigated and widely used due to their peculiar physical–chemical properties [43,44,45,46]. LDHs are structurally similar to brucite Mg(OH)_2_ and crystallize in a layer-type lattice. They can be generally represented by the chemical structure $[M_{1-x\;}^{\left(II\right)}M_{x\;}^{\left(III\right)}{\left(\mathrm{OH}\right)}_{2}{]}^{x+}\;[A_{x/m\;}^{-}]\cdot nH_{2}O$, where *M*^(*II*)^ is a divalent metal cation (Mg, Mn, Fe, Co, Ni, Cu, Zn, Ga), *M^(III)^* is a trivalent metal cation (Al, Cr, Mn, etc.), and *A*^−^ represents an interlayer anion (carbonate, nitrate, etc.). The addition of LDH has been extensively reported, and it has been demonstrated that anionic platelets have a beneficial effect on the water retention capacity, thermal resistance, mechanical strength, and proton conductivity of the nanocomposite PEM [47,48,49]. However, the influence of the LDH particles on the methanol permeability of sPEEK membrane has, to date, not yet been clarified.

To fill this gap, we propose the preparation of sPEEK-LDH nanocomposite membranes as a simple and low-cost method for enhancing the transport properties of the sPEEK-based electrolytes while strongly enhancing the methanol resistance. Accordingly, a Mg/Al LDH (Mg^2+^/Al^3+^ metal ratio of 2:1 and ${\mathrm{NO}}_{3}^{–}$ as interlayer anions) was synthetized and homogeneously dispersed in the polymer matrix. The resulting membranes were characterized for their thermo-mechanical properties by DMA and their dimensional stability was evaluated under various methanol concentrations. NMR techniques (diffusometry and relaxometry) were used to investigate water and methanol molecular dynamics, while electrochemical impedance spectroscopy was used to assess the proton conductivity. Acting as a physical crosslinker, LDH platelets massively decreased the methanol mobility while simultaneously promoting the formation of an extremely well-connected path for proton transport.

## 2. Materials and Methods

### 2.1. Materials

Polyether ether ketone polymer (PEEK, Victrex 450PF) was purchased from ICI (London, UK) and dried in a vacuum oven at 100 °C for 24 h before use. N,N-dimethylacetamide (DMAc), NaOH (0.1 M, volumetric standard), and sulfuric acid (95–98 wt%) were purchased from Sigma-Aldrich (Sigma-Aldrich, Milan, Italy) and used as received. Ion Power (Ion Power Inc., New Castle, DE, USA) supplied us with a Nafion 212 membrane which was chemically activated using the process previously reported by us [50].

### 2.2. Synthesis of Layered Double Hydroxides (LDH)

Layered double hydroxide material (Mg^2+^/Al^3+^_${\mathrm{NO}}_{3}^{–}$) was synthesized in accordance to the procedure reported elsewhere [51]. The Mg^2+^ and Al^3+^ nitrate salts were co-precipitated under inert atmosphere (N_2_ gas flow) in an aqueous solution of NaOH (2.5 M) until the pH of the solution reached 10. The solution was left under stirring for 6 h at 60 °C, and then centrifuged at 5000 rpm to recover the LDH particles. Finally, the precipitate was washed several times with distilled water and dried in the oven at 80 °C for one day. The metal ratio (Mg^+^:Al^+^) was fixed to 2:1.

### 2.3. Sulfonation of Polyether Ether Ketone 

The synthesis of sulfonated PEEK was carried out according to the methodology proposed by Simari et al. [29]. PEEK was dissolved in concentrated H_2_SO_4_ at room temperature under vigorous stirring until a homogeneous solution was obtained. Thereafter, the temperature was increased to 40 °C and the reaction was left for 5 h. To quench the reaction, the resulting polymer solution was slowly poured into ice-cold distilled water (4 °C) under continuous stirring resulting in the precipitation of sPEEK in powder form. The polymer flakes were recovered by filtration, vigorously washed with deionized water (until pH 6–7), and then heated at 60 °C until dryness.

### 2.4. Preparation of Membranes

Bare sPEEK membrane was obtained by dissolving 150 mg of polymer in DMAc (10 wt% solution) at room temperature. The solution was then cast on a glass plate and heated at 60 °C until the solvent completely evaporated. In the case of sPEEK-LDH composite membranes, the LDH material was directly dispersed in the polymer solution (10 wt% of sPEEK in DMAc) by alternating vigorous mechanical stirring with ultrasonication. The fine dispersion was cast on a glass plate and dried for 18 h at 55 °C. Membranes at different LDH loading were prepared (1, 3, and 5 wt%), and the resulting nanocomposite membranes were designated as sPL_x_, where x represents the mass percentage of LDH with respect to the polymer. Finally, both pristine and composite membranes were converted into the acid form by soaking them in 0.5 M H_2_SO_4_ solution followed by washing several times with distilled water to remove any residual acid (i.e., until the pH of the water was 6–7). The average thickness of the membranes ranged between 50 and 55 μm.

### 2.5. Characterization Techniques

The ion exchange capacity (IEC) was determined by titration method [52], while solution uptake was calculated by considering the weight variation between dry and wet states. Activated membranes were put in 2 M NaCl solution for 16 h to exchange the hydrogen ions (H^+^) with Na^+^, and the released H^+^ was back-titrated with 0.1 M NaOH solution using phenolphthalein (C₂₀H₁₄O₄) as the indicator. *IEC* was calculated in meq g^−1^ according to Equation (1).

(1) $$IEC\;\left(\mathrm{meq}\cdot g^{-1}\right)=\frac{M_{\left(NaOH\right)}\;V_{\left(NaOH\right)}}{W_{dry}}$$

Solution uptake was calculated by considering the weight difference in wet and dry states. Briefly, the membranes were cut first into a rectangular-shaped piece and then immersed in the swelling solution at room temperature for 24 h. Subsequently, samples were recovered, their surface rapidly blotted with a tissue paper, and the weight immediately measured (*M_wet_*). To get the dry weight (*M_dry_*) of these membranes, they were put in an oven for drying at 60 °C for 16 h and then their weight was measured. Solution uptake was assessed both in pure water and in aqueous methanol solution (concentration ranging between 1 M and 5 M). Consequently, the solution uptake was calculated according to Equation (2):

(2) $$∆U\;\left(\%\right)=\frac{M_{wet}-M_{dry}}{\;M_{dry}}*100$$

X-ray diffraction (XRD) measurements were performed using the Cu-Kα radiation of a Bruker Axis Diffractometer/Reflectometer (D8) equipped with a Dynamic Scintillation Detector, NaI, and with a Göbel mirror. Spectra were collected at room temperature in the 2θ range from 5° to 40°, in steps of 0.03°, and the counting time was 1 s/step [50].

Dynamic mechanical analysis (DMA) was conducted on rectangularly shaped samples (10 mm × 30 mm) using a Metravib DMA/25 analyzer equipped with a shear jaw for film clamping [53]. The dynamic stress had an amplitude equal to 10^−4^ Pa while the frequency was set at 1 H. The viscoelastic properties of the material were assessed in the temperature range of 25–180 °C, with a heating rate of 2 °C min^−1^.

The ^1^H-NMR measurements were performed on a Bruker AVANCE 300 wide bore spectrometer working at 300 MHz on ^1^H and equipped with a Diff30 Z-diffusion 30 G/cm/A multinuclear probe with substitutable RF inserts. The self-diffusion coefficients (D) of water and methanol were measured by the pulsed field gradient stimulated-echo (PFG-STE) technique [54]. In these experiments, δ and Δ were kept at 0.8 and 8 ms, respectively, while g was varied between 100 and 900 G cm^−1^. Based on the very low standard deviation of the fitting curve and repeatability of the measurements, the uncertainties in D values were calculated to be circa 3%. Longitudinal relaxation time (T_1_) values of water and methanol were instead obtained by the inversion recovery sequence (π-τ-π/2). Both D and T_1_ measurements were conducted by increasing the temperature in the range of 20–130 °C with steps of 20 °C. Samples were equilibrated for approximately 20 min at each temperature. The use of deuterated solvents allowed the ^1^H-NMR signal of water to be discriminated from that of methanol. Details about the preparation of the NMR sample are provided elsewhere [55].

To measure through-plane proton conductivity, samples were cut into a circular shape (Ø = 10 mm) sandwiched between two circular pieces of conductive carbon paper (Ø = 8 mm) and placed in a homemade two-electrode cell connected with a fuel cell test hardware (850C, Scribner Associates, Inc., Southern Pines, NC, USA). A humidification system (Fuel Cells Technologies, Inc., Albuquerque, NM, USA) was used to control the relative humidity (RH, %) by mixing the dry and wet gases. Impedance spectra were recorded on a PGSTAT 30 potentiostat/galvanostat/FRA at OCV over the frequency range of 1 Hz−1 MHz by applying an oscillating potential of circa 10 mV. The resulting impedance data were analyzed by Metrohm Autolab NOVA software and the electrolyte resistance (Rel) was extracted from the high-frequency intercept on the real axis in the Nyquist plot. Impedance measurements were carried out between 30 and 120 °C at 90% RH. Proton conductivity, σ (S/cm) was calculated using the following Equation (3).

(3) $$\sigma \;\left({S\;\mathrm{cm}}^{-1}\right)=\frac{l}{\;R_{el}*A}$$

where *l* is the membrane thickness, *R_el_* is the electrolyte resistance, and *A* is the active area.

The chemical stability of the PEMs was measured at 80°C using Fenton’s reagent (5% H_2_O_2_ and 1 ppm Fe_2_SO_4_). Activated membranes were dried, weighted (*M_before_*), and finally immersed into the Fenton’s solution at 80 °C for up to 48 h. For each interval time, the samples were rinsed with excessive water to stop the degradation process, dried, and weighted again (*M_after_*). The residual weight of the membrane was calculated according to Equation (4):


(4) $$Residual\;weight\;\left(\%\right)=\frac{M_{after}}{\;M_{before}}*100$$

## 3. Results and Discussion

### 3.1. Morphological and Thermo-Mechanical Characterization

**Figure 1:**
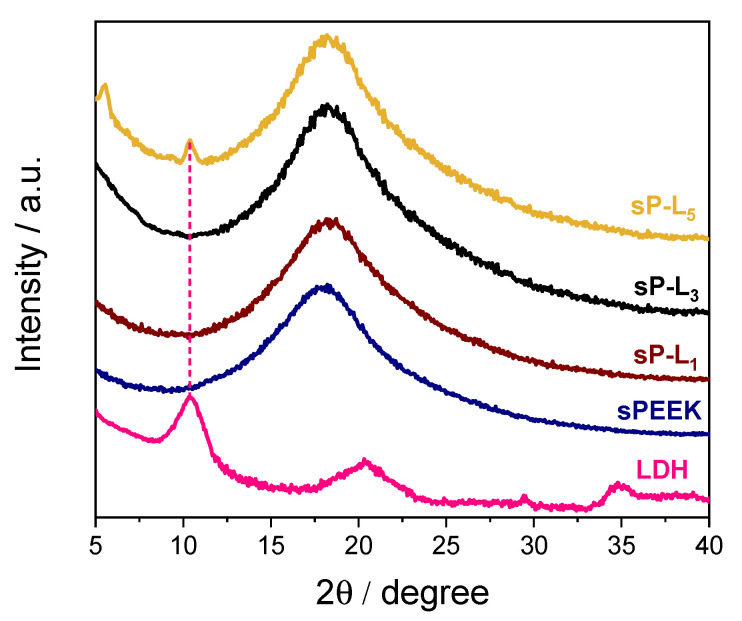
XRD patterns of Mg/Al-LDH powder, sPEEK, and sP-L_x_ nanocomposite membranes.

Figure 1 compares the XRD profile of LDH powder with those of pristine sPEEK and composite membranes at different filler loadings. The powdered Mg/Al_${\mathrm{NO}}_{3}^{–}$ sample shows the typical features of layered materials, with a main diffraction peak at 2θ = 10.1° corresponding to a basal spacing (d_003_) of ca. 9 Å [56]. The XRD pattern of sPEEK is characterized by the presence of a broad band covering the 2θ range of 12–30 degrees and without any crystallinity, indicating the polymer has a completely amorphous structure. Despite the presence of the LDH, the XRD profiles of both sPL_1_ and sPL_3_ nanocomposite membranes are completely superimposable to that of bare sPEEK. This suggest the LDH platelets lost their staking in these nanocomposite membranes [57]. Being negatively charged, sPEEK can establish favorable electrostatic interactions with LDH platelets (which are positively charged), thus promoting their complete exfoliation within the polymer matrix. Conversely, two additional weak and sharp diffraction peaks can be seen in the spectra of the sPL_5_ membrane. While the peak at 2θ ≈ 10° clearly indicates the presence of some LDH particles, the signal at 5.5° likely results from polymer intercalation within the interlayer space of LDH platelets [57].

**Figure 2:**
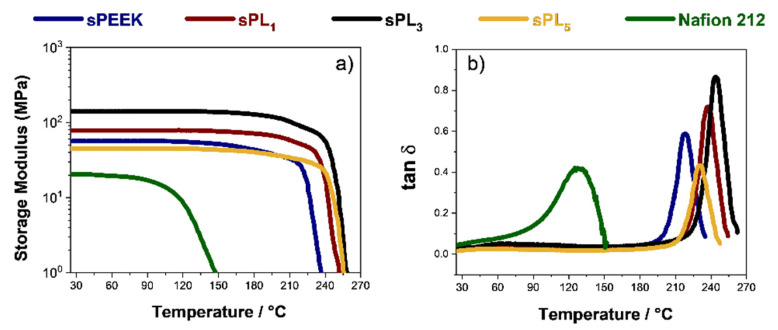
(**a**) Storage modulus and (**b**) tan δ of the sPEEK-based nanocomposite membranes as a function of the temperature.

To gain insight on the thermo-mechanical performance of the sPEEK-based membranes, extensive DMA characterization was carried out. The temperature evolution of the storage modulus (E’) and dumping factor (tan δ) plot for the prepared membranes is shown in Figure 2a,b, respectively. The viscoelastic behavior of the commercial Nafion 212 membranes was also plotted for comparison. Clearly, all the sPEEK membranes exhibit a superior mechanical performance compared to the Nafion benchmark. Indeed, even the storage modulus of the bare sPEEK is remarkably higher than that of Nafion, i.e., 56 MPa vs. 20 MPa, respectively. It is worth noting that E’ progressively increases with increasing filler content until 3 wt%. In fact, the beneficial interactions between the polymer chains and the LDH platelets provide an impressive mechanical robustness for the sPL_3_ membrane, which shows a storage modulus of ca. 150 MPa, i.e., almost one order of magnitude higher than that of Nafion 212. Furthermore, the LDH addition also results in a significant extension of the thermal resistance. Indeed, the storage moduli remains quite constant until almost 210 °C, indicating the nanocomposite membranes are able to withstand very high operating temperatures without dimensional changes and physical deterioration. The relationship between thermal resistance and LDH content is better elucidated by the temperature evolution of the dumping factor plot illustrated in Figure 2b. A single peak is clearly visible in the tan δ profiles of all the investigated membranes. The latter is generally ascribed to the α-transition (T_g_) of the ion-conductive clusters. However, this transition progressively shifts toward higher temperatures with increasing filler content. Accordingly, the sPL membranes can successfully withstand higher working temperatures. Similarly, the loss tangent value also increases, thus indicating the nanocomposites better dissipate undesirable mechanical solicitation. In this regard, the sPL_3_ membrane combines a high Tg (245 °C) with a high dumping capacity (close to 1), which are both beneficial features for polymer electrolytes to be applied in DMFCs [40,58].

### 3.2. Ion Exchange Capacity (IEC) and Dimensional Stability

**Figure 3:**
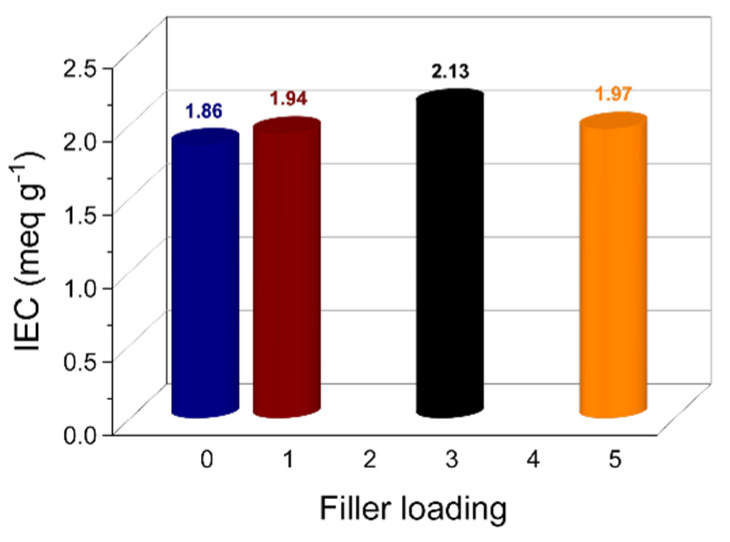
Ion exchange capacity of the sPEEK-based membranes at different filler loadings.

The ion exchange capacity (IEC) relates to the total number of functional groups available for ion transfer and thus for proton conduction [59]. Accordingly, it is a crucial parameter for polymer electrolyte membranes. Figure 3 illustrates the IEC variation as a function of the filler content. The pristine sPEEK membrane shows a quite high IEC value, i.e., 1.86 meq g^−1^, which further increases after the introduction of the LDH platelets, reaching the maximum value of 2.13 meq g^−1^ for sPL_3_. Due to the charged nature of the anionic nanoclays, their complete exfoliation within the sPEEK matrix results in a remarkable increase in the number of hydrophilic sites involved in the ion exchange. However, higher filler content results in a decrease in the IEC. The XRD analysis, in fact, proved that some of the LDH particles keep their staking in the sPL_5_ membrane. This trivially reduces the number of polar groups available for proton conduction.

**Figure 4:**
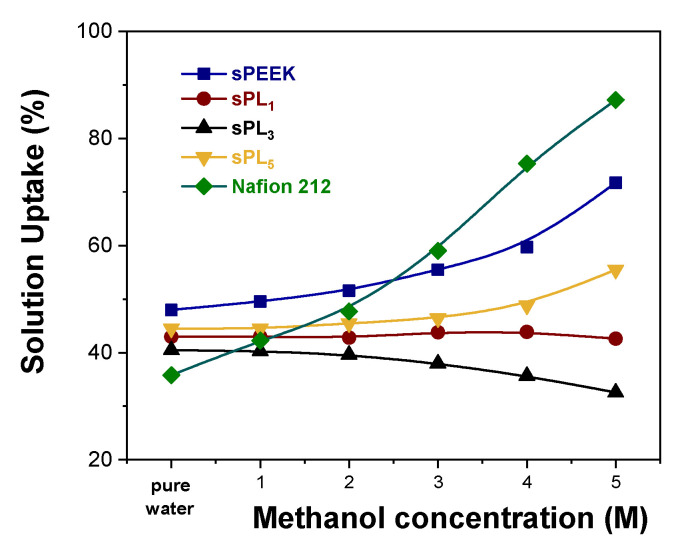
Solution uptake vs. methanol concentration for Nafion 212 and sPEEK-based membranes.

The number of hydrophilic sites also impacts on the dimensional stability of the electrolyte membrane. Typically, the higher the IEC capacity, the larger the swelling into polar solvents, such as water and methanol [60]. In this regard, an adequate amount of water molecules is obviously required to achieve good proton conductivity, but excessive swelling generally results in rapid MEA degradation, a poor mechanical performance, and the permeation of non-desirable components through the membrane, which de facto limits its practical application in DMFCs. Accordingly, the solution uptake (ΔU, %) for the sPEEK-based membranes was measured as a function of the methanol concentration. Data are illustrated in Figure 4 in comparison with Nafion 212, which is used as a benchmark. Compared to Nafion 212, sPEEK exhibits a better dimensional stability under increasing methanol concentration. It is well known that methanol acts as a plasticizer on the Nafion polymer chains. A higher methanol concentration produces larger hydrophilic channels, which in turn results in membrane overswelling (more than 90 wt% uptake) and thus in the formation of methanol crossover clusters. Conversely, sPEEK has an inherently lower affinity toward MeOH. This preserves the dimension of its ionic cluster, thus limiting solution uptake. It is worth noting that the introduction of the LDH platelets further enhances the methanol resistance of the resulting membranes. As mentioned above, LDH nanoparticles (which are positively charged) experience a strong electrostatic interaction with negatively charged sulfonic groups anchored to the polymer chains of sPEEK. Due to the very high charge density of the LDH material, we can hypothesize that one LDH lamella might simultaneously interact with more than one –SO3H functionalities, thus acting as a physical crosslinker. Consequently, the LDH platelets prevent any alteration in the hydrophilic channels, thus providing impressive dimensional stability over a wide range of methanol concentrations.

### 3.3. NMR Investigation

**Figure 5:**
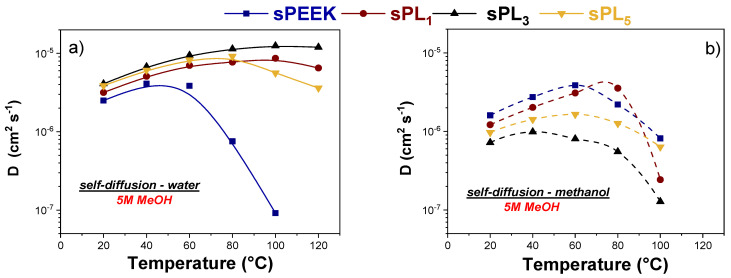
(**a**) Self-diffusion coefficients of water and (**b**) methanol, in 5 M solution confined in sPEEK and sPL membranes, from 20 up to 130 °C.

Due to the outstanding dimensional resistance of the sPEEK-LDH nanocomposite membranes, we attempted to clarify the molecular dynamics of water and methanol into swollen electrolytes by the PFG-NMR technique. The NMR signals originating from water and methanol were decoupled by using deuterated solvents: a mixture of CD_3_OD/H_2_O and CH_3_OD/D_2_O was used to selectively investigate the molecular mobility of water and methanol, respectively [42]. In this regard, Figure 5 illustrates the temperature evolution of the self-diffusion coefficients of water (D_wat_) and methanol (D_met_) inside the sPEEK-based membranes upon swelling into a 5 M methanol solution. We would like to point out that membranes were also tested under a lower concentration, i.e., a 2 M methanol solution. Under this condition, however, al the sPEEK-based membranes exhibited a similar behavior, with D_wat_ clearly exceeding D_met_. The evidence further confirms that sPEEK-based membranes inherently provide a reduced methanol permeability compared to Nafion. For the latter, in fact, the methanol diffusivity overcomes the water diffusivity starting from relatively-low temperatures and low methanol concentrations [61]. Turning the attention on the measurements at the 5 M methanol concentration, some crucial aspects clearly emerge:

(i) In the case of pristine sPEEK, D_wat_ and D_met_ were almost comparable until 60 °C; then, the methanol diffusivity started significantly exceeding the water diffusivity. This means the synergy between a high methanol concentration and a relatively high temperature has a detrimental effect on the methanol resistance of pure sPEEK
(ii) The introduction of the LDH particles has a beneficial effect on the water self-diffusion coefficients while producing a remarkable reduction in the methanol mobility. Consequently, the water diffusivity is always higher than the methanol diffusivity for all the nanocomposite membranes and for all the temperature range investigated.
(iii) The sPL_3_ membrane showed the best performance among the investigated membranes. Indeed, this membrane showed the highest discrepancy, combining outstanding water transport properties, i.e., a high water mobility even under high temperatures, with an impressive methanol resistance. In fact, at 100 °C, D_wat_ in sPL_3_ is more than two orders of magnitude higher than D_met_, i.e., 1.25 × 10^−5^ cm^2^ s^−1^ vs. 9.13 × 10^−8^ cm^2^ s^−1^, respectively.

The evidence suggests the homogeneous dispersion and complete exfoliation of the LDH platelets is able to increase the methanol resistance of the resulting membranes, likely due to an increase in the tortuosity of the methanol diffusivity path, while preserving adequate water mobility. 

**Figure 6:**
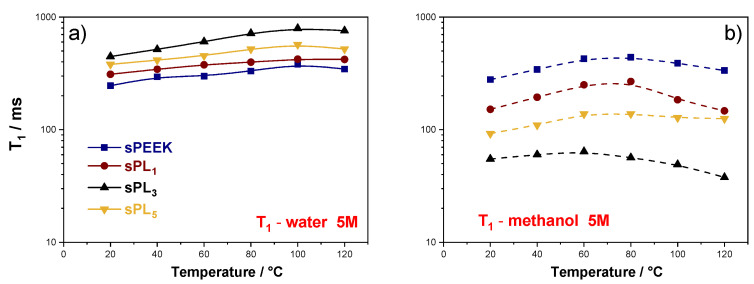
Temperature evolution, in the range of 20–130 °C, for the spin-lattice relaxation times (T1) of (**a**) water and (**b**) methanol measured on sPEEK and sPL membranes swollen in 5 M solution.

The analysis of the NMR longitudinal relaxation times (T_1_), reported in Figure 6, provides additional insights about the microscale mobility of the two solvents. Compared to the self-diffusion coefficient, T_1_ values are more sensitive to short range motions, including translation and rotation of the molecules on a time scale comparable to the reciprocal of the NMR angular frequency (~1 ns). Typically, the higher the T_1_ values, the easier the molecular motions. It is possible to see in Figure 6a that the relaxation times for water increases after the addition of the LDH particles, reaching the highest values in the sPL_3_. Such higher T_1_ values, which relate to a higher degree of freedom, are highly desired in the case of proton-conducting electrolytes. Indeed, since water molecules are directly involved in the proton transport mechanisms, either by vehicular or Grotthuss ones, a larger local mobility generally results in a better proton conductivity. Conversely, the methanol T_1_ values in the nanocomposite membranes are considerably lower than those of pristine sPEEK (see Figure 6b), indicating that, also at the molecular scale, the methanol mobility is massively hampered by the presence of the LDH lamellae. Accordingly, it can be safely stated the nanoplatelets are able to massively decrease the methanol crossover by obstructing the diffusional path responsible for methanol migration via electro-osmotic drag [62].

### 3.4. Proton Conductivity (σ) and Oxidative Stability

**Figure 7:**
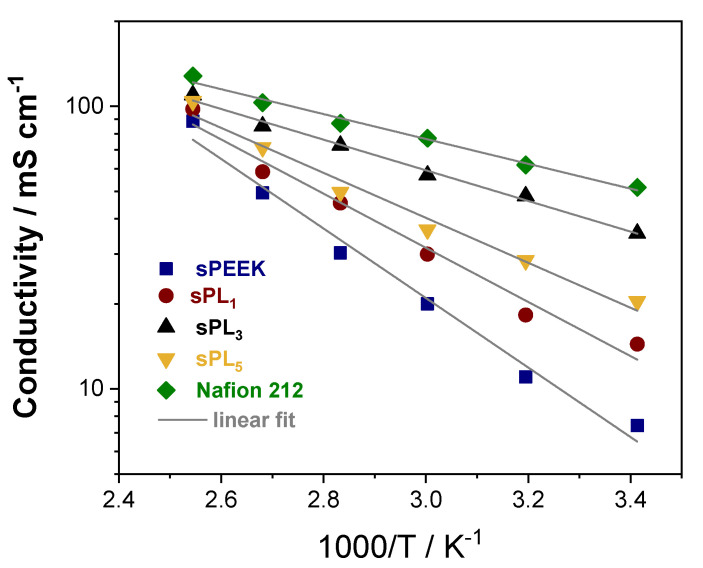
Temperature-dependence of the proton conductivity at 90% RH. Nafion 212 is also reported for comparison.

In addition to the low methanol permeability, polymer electrolyte membranes for DMFCs have to ensure a satisfactory through-plane conductivity (σ). The latter, in fact, is typically considered as one of the key factors since it can affect the final performance of the device. To definitely assess the suitability of the sPL samples in real fuel cell systems, their through-plane conductivity (σ) was measured in the temperature range between 20 and 120 °C at 90% RH, and the results are illustrated in Figure 7. The conductivity of Nafion 212 is also reported for comparison. Despite having a higher IEC, i.e., 1.86 meq g^−1^ for sPEEK vs. 0.94 meq g^−1^ for Nafion 212, the σ for pristine sPEEK is distinctly lower. Compared to the Nafion benchmark, the sPEEK microstructure is characterized by smaller, narrower, and less interconnected ionic clusters with a larger number of dead-end “pockets”. Also, the average distance between adjacent sulfonic acid groups is larger [63]. The features have, obviously, a detrimental effect on the conductivity performance of the bare sPEEK. Nonetheless, the addition of the anionic clay platelets results in an impressive improvement of the proton conductivity. Once again, the best performance was attained on membranes at a 3 wt% loading. Indeed, the sPL_3_ membrane exhibited a proton conductivity of 35 mS cm^−1^ at 20 °C and 110 mS cm^−1^ at 120 °C, almost attaining the performance of Nafion 212. This suggests the LDH lamellae provide additional sites that are directly involved in the proton conduction via the Grotthuss mechanism. The latter assumption is further corroborated by the analysis of the relative activation energies (Ea) for proton conduction calculated from the Arrhenius plots and reported in Table 1. It is possible to see that the Ea for the sPL_3_ sample is almost superimposable to the activation energy of Nafion 212, being 10.4 kJ mol^−1^ and 9.9 kJ mol^−1^, respectively. In fact, the anionic lamellae are able to fill the gap between adjacent –SO_3_^−^ groups of sPEEK, thus enabling the formation of an extensive and extremely connected pathway, thus boosting the proton conduction.

**Figure 8:**
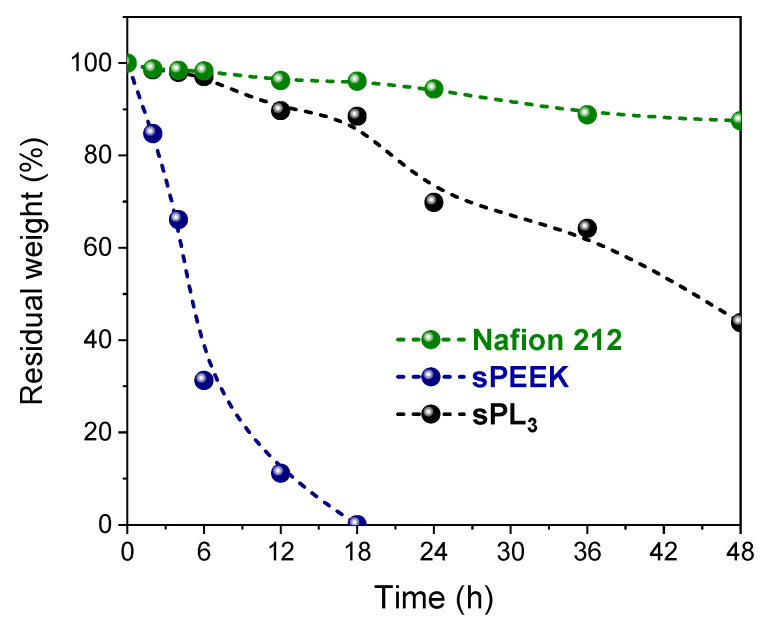
Weight variation (%) for Nafion 212, sPEEK, and sPL_3_ membranes after exposition to the Fenton reagent solution at 80 °C over 48 h.

Finally, the chemical oxidative resistance of the sPEEK membranes was studied by Fenton’s test at 80 °C in terms of weight loss over 48 h and the results are illustrated in Figure 8, in comparison with Nafion 212. The latter exhibits the best oxidative stability, with more than 90% of its weight retained after 48 h of degradation with the Fenton reagent. Due to its perfluorurate backbone, Nafion 212 possesses a higher resistance toward the radical attack responsible for polymer degradation during Fenton’s test [64]. Conversely, rapid and massive weight losses were observed for pristine sPEEK, which failed to withstand the radical attack even up to 18 h of continuous testing. It is evident that the introduction of the LDH nanoplatelets remarkably increases the chemical stability of the resulting membrane. Compared to parental sPEEK, sPL_3_ exhibits a slower degradation rate and can maintain 44% of its weight after 48 h of degradation. It is likely that the strong electrostatic interaction of LDH nanoplatelets with sulfonic acid groups increases the steric hindrance for the radical attack in the hydrophilic positions, thus enhancing the oxidative stability.

## 4. Conclusions

In this preliminary study, nanocomposite membranes based on sulfonated poly(ether ether ketone) and layered double hydroxides have been investigated as inexpensive and good performing polymer electrolyte membranes for DMFCs operating under high methanol concentrations. In particular, composite membranes at three filler loadings (1, 3, and 5 wt% with respect to the polymer) were prepared and assessed for their structural, thermo-mechanical, dimensional, and transport properties. The homogeneous dispersion and complete exfoliation of the anionic clay platelets has a beneficial effect on the mechanical and thermal resistance of the resulting electrolytes, which are thus able to successfully withstand high operating temperatures and severe mechanical solicitations. The swelling tests, performed under various methanol concentrations, revealed that the dimensional stability of sPL nanocomposite membranes is remarkably higher compared to both pure sPEEK and Nafion 212 samples. Clearly, the LDH nanoplatelets also impact the transport properties of the PEMs. Compared to pristine sPEEK, the methanol mobility is remarkably lower whilst the water mobility remains very high. In a nutshell, LDH platelets act as a physical crosslinker between adjacent sulfonic groups of the sPEEK backbone. This increases the tortuosity in the diffusion path for methanol permeation, but promotes the formation of a highly connected network that promotes the proton conduction. In fact, the sPL membrane at 3 wt% of filler with respect to the polymer was able to achieve a proton conductivity of 110 mS cm^−1^ at 120 °C and 90% RH, which was almost comparable to that of the Nafion benchmark (127.9 mS cm^−1^). The ease of preparation, impressive methanol resistance, good ion conductivity, and satisfactory oxidative resistance of the sPL_3_ membrane hold promise for its successful application in DMFCs.

## Figures and Tables

**Table 1:** Proton conductivity and activation energy (Ea) for Nafion and sPEEK-based membranes.

Membranes	Proton Conductivity—σ (mS cm^−1^)		Ea (kJ mol^−1^)
	60 °C	120 °C	
sPEEK	19.97	88.59	19.4
sPL_1_	29.9	97.7	18.4
sPL_3_	60.9	109.5	10.4
sPL_5_	36.6	104.1	15.2
Nafion 212	77.1	127.9	9.9

## Data Availability

Data is contained within the article.
